# Forgotten left bronchial foreign body spontaneously expelled during chest physiotherapy: a pediatric case report

**DOI:** 10.3389/fmed.2026.1784458

**Published:** 2026-04-02

**Authors:** Hina Ayesha, Rabia Noor, Maryam Tariq, Zuha Tariq, Muhammad Arham, Salomon Izere

**Affiliations:** 1 Department of Pediatrics, Aziz Fatimah Medical and Dental College, Faisalabad, Pakistan; 2 Aziz Fatimah Hospital, Faisalabad, Pakistan; 3 Department of Medicine, Aziz Fatimah Medical and Dental College, Faisalabad, Pakistan; 4 Department of Medicine, Allama Iqbal Medical College, Lahore, Pakistan; 5 Department of Medicine, Aziz Fatimah Hospital, Faisalabad, Pakistan; 6 Department of General Medicine and Surgery, University of Rwanda College of Medicine and Health Sciences, Kigali, Rwanda

**Keywords:** chest physiotherapy, chronic cough, coughing, foreign body, pediatric

## Abstract

Foreign body aspiration is common in pediatric setting and in chronic cases such patients often present with clinical features similar to infective respiratory etiologies. This is the case of a forgotten left bronchial foreign body in a 5-year-old girl, who presented with worsening of recurrent cough and fever. She was previously being treated as case of bronchial asthma and foreign body was missed on the imaging as well. It was subsequently expelled by chest physiotherapy. The case highlights the importance of having foreign body aspiration (FBA) as a differential in cases of chronic cough, even without any history of choking, to avoid diagnostic challenge. Moreover, spontaneous expulsion of foreign body was incidental clinical event and is not substitute for standard clinical care.

## Introduction

Foreign body aspiration (FBA) more common among males and in the right bronchus, usually presents with cough, stridor, wheeze and choking (1, 2). Such events are preceded with history of choking whereas, at times patient lacks both history and bronchial cut off sign on imaging and are referred to as occult tracheobronchial foreign bodies’ (OTFB), having 4.4% incidence (1, 3).

Globally, the age-standardized incidence of FBA among children under 5 years in 2019 was ~109.6 per 100,000, with a disability-adjusted life-year (DALY) rate of ~317.9 per 100,000.

**Figure 1:**
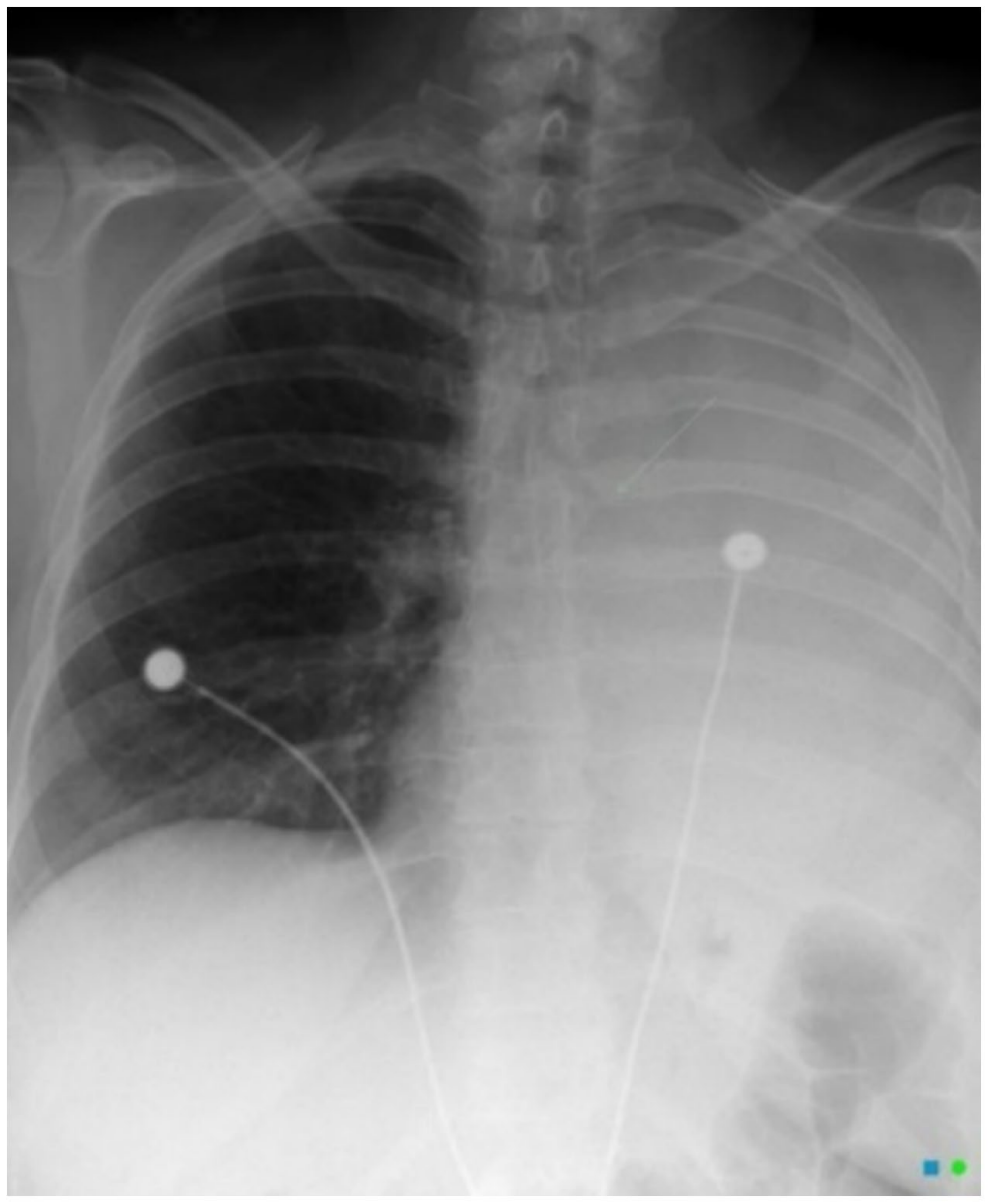
Representative imaging of bronchial occlusion in 44 year old female. Image courtesy of Dr. Chris O’Donnell, Radiopaedia.org, rID: 76649 (used under CC BY-NC-SA).

Causes of missed OTFB on radiology include, more elastic and smaller airway, foreign bodies in subglottic area, artefacts due to improper sitting and setting, thin-flaky and low-density materials (4). Typical radiographic picture known as bronchial cut-off sign as reported by O’Donnell C. in 44 year-old female is seen in the case of bronchial obstruction, as shown in Figure 1 (5). It is recommended to go for bronchoscopy in all pediatric cases with chronic pneumonia having consolidation, emphysema and atelectasis on imaging to rule out OTFB, despite absence to bronchial cut-off signs on imaging (1).

Immediate recognition and intervention are crucial to prevent complications such as asphyxia, pneumonia, or bronchiectasis or even cardiac arrest (6). In such cases the X-ray chest is considered the best first line investigation, serving as the screening tool to guide physicians about the suspected etiology (7). The prognosis of FBA significantly improves with early detection and appropriate management. Appropriate management includes encouraging cough and back blows in mild and severe cases, respectively. Chest compressions should be reserved for life-threatening cases. Laryngoscopy, bronchial isolation and bronchoscopy will follow the general maneuvers (8).

In such cases, fiberoptic flexible bronchoscopy is considered one of the safest and first line modality with high negative predictive value (9).

Rigid bronchoscopy remains the gold standards for both diagnosis and therapeutic removal of the foreign body, despite being invasive procedure (10, 11). Rigid Bronchoscopy has the highest success rate for removal of most of the aspirated foreign bodies in most of the case with a median of 16 min procedure time (11). Delayed treatment can lead to severe complications, including chronic lung infections, bronchial stenosis, and in severe cases, death.

It is very important to confirm a diagnosis of Foreign Body Aspiration (FBA) as it may mimic and hide other serious conditions like Lung Neoplasms and the delay in recognition between the two may lead the patient towards fatal consequences (11).

## Case presentation

Previously healthy, the 5-year-old Punjabi-Pakistani girl presented in the outpatient department with the presenting complaint of recurrent cough that worsened for 1 month. The girl had a history of frequent episodes of cough associated with sputum production and shortness of breath over a period of 1 year. Cough was productive, with 1–2 teaspoonfuls of whitish mucoid color, devoid of blood, seasonal or diurnal variation, but associated with episodes of wheeze and shortness of breath.

Over the course of 1 year, she had been visiting multiple physicians, including pediatricians and adult pulmonologists, and was misdiagnosed as a case of bronchial asthma and treated accordingly. This led to fragmented medical history during this period leading to unclear timeline in this 1 year period. She was admitted to the pediatric ward with worsening symptoms. Her General Physical Examination showed no pallor, cyanosis, edema, clubbing, or jaundice. She was well-oriented in time and space yet lethargic. Upon auscultation, bilateral rhonchi and rales were appreciated. Her abdomen was soft and nontender, and no organomegaly was seen. Bowel sounds were also normal. She was delivered by C-section at the 36th week of gestation and was breastfed for 2 months along with formula milk. Developmental milestones were normal. She had no history of contact with birds or animals at home. Moreover, the familial history of Tuberculosis or asthma was also absent. She was vaccinated as per National Expanded Program on Immunization. She had no known personal allergy. She had a history of eating disorder of consuming non-food material (PICA) including eating gravel and dirt that parents confessed after the expulsion of foreign body. She was put on antibiotics, steroids, bronchodilators, nebulization, and chest physiotherapy. The mother was guided to perform chest physiotherapy on the patient.

## Investigations

**Figure 2:**
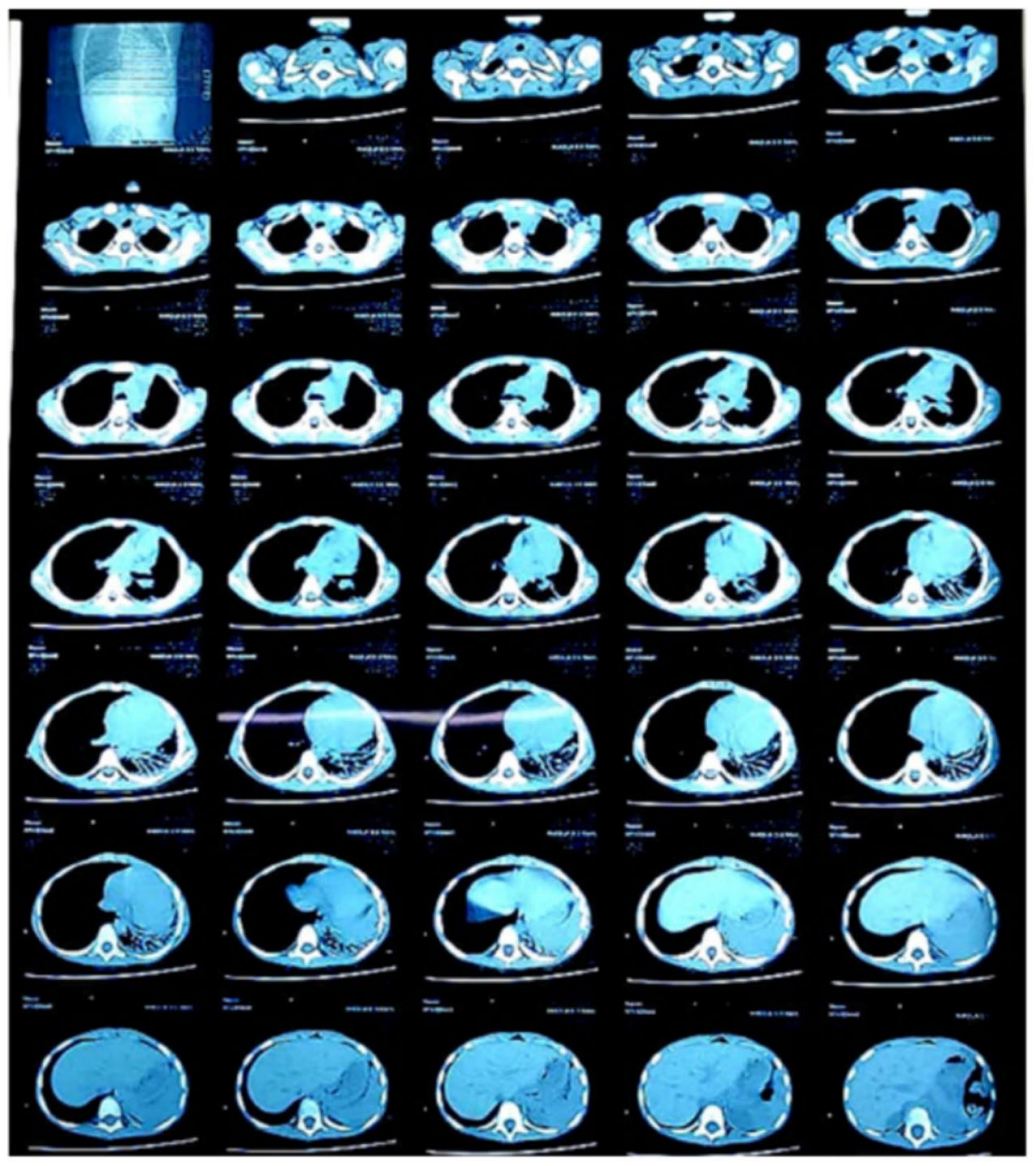
Chest high resolution computed tomography conducted a month before presenting to our hospital—showing a patch of consolidation with internal air bronchograms and sub-segmental collapse in the left lower lobe with mild ipsilateral cardio-mediastinal shift.

During her treatment with other physicianschest X-ray was advised, a month before presentation to our hospital, which raised suspicion of collapse, upon which High-Resolution Computed Tomography (HRCT) was advised during the pre-admission period (Figure 2). It revealed a patch of consolidation with internal air bronchograms and subsegmental collapse in the left lower lobe with mild ipsilateral cardio-mediastinal shift. The radiologist suggested post-infective etiology, and bronchiectasis of the lower lobe of the left lung was suspected.

**Figure 3:**
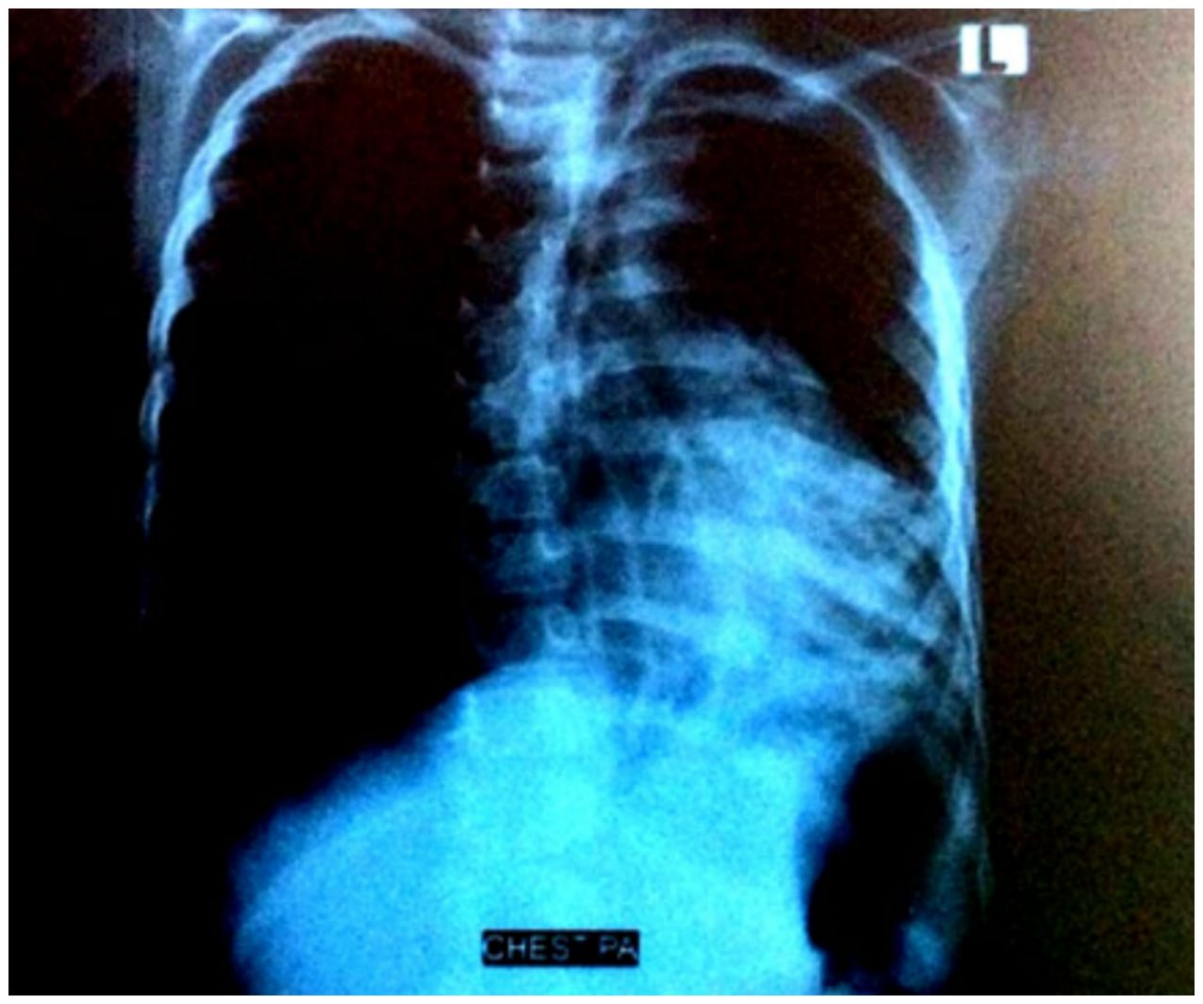
Chest X-ray on first day of admission showing right lung hyperinflation (pre-expulsion).

**Figure 4:**
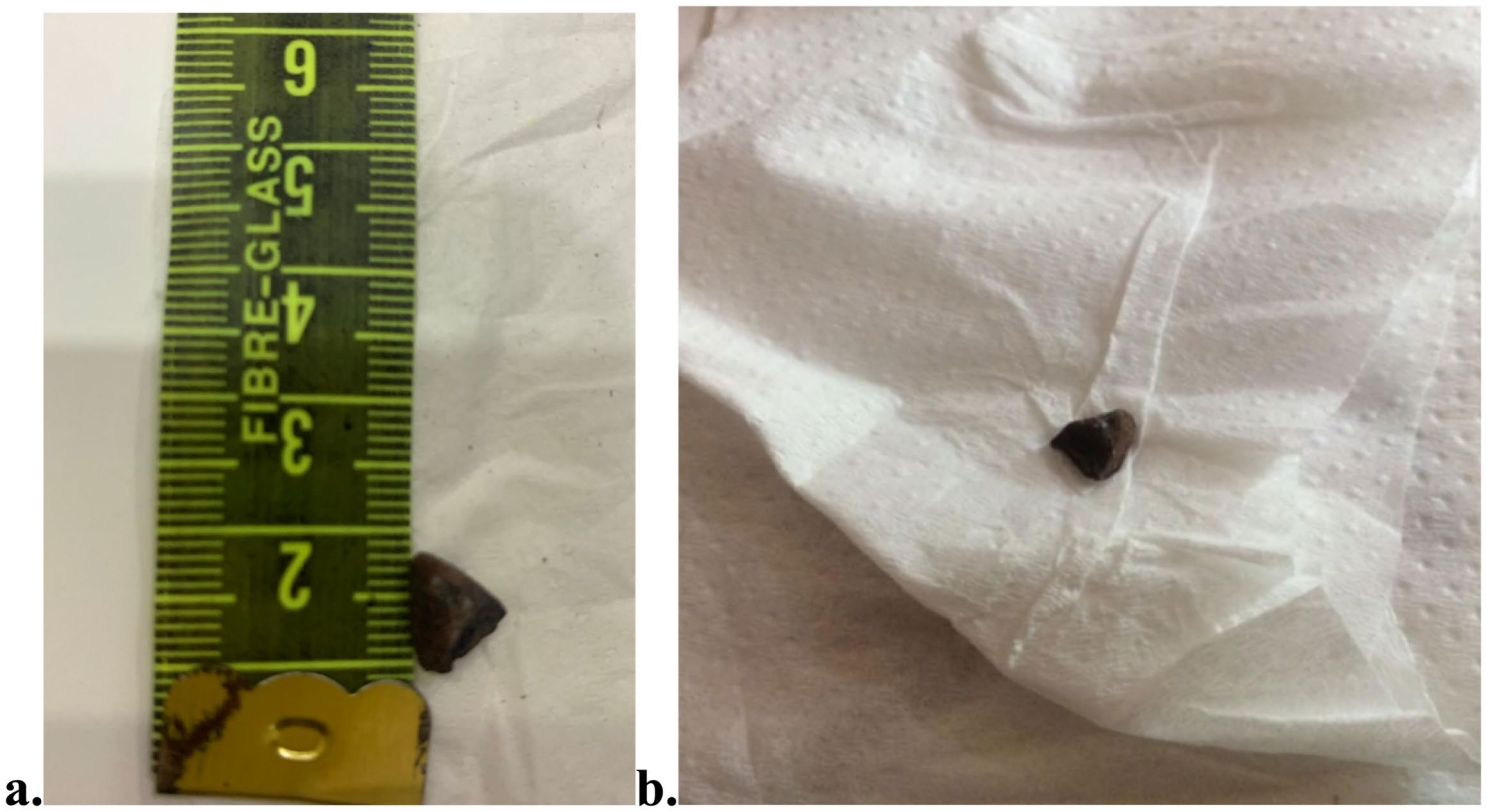
**(a,b)** A stone of around 9 mm was coughed out during chest physiotherapy.

**Figure 5:**
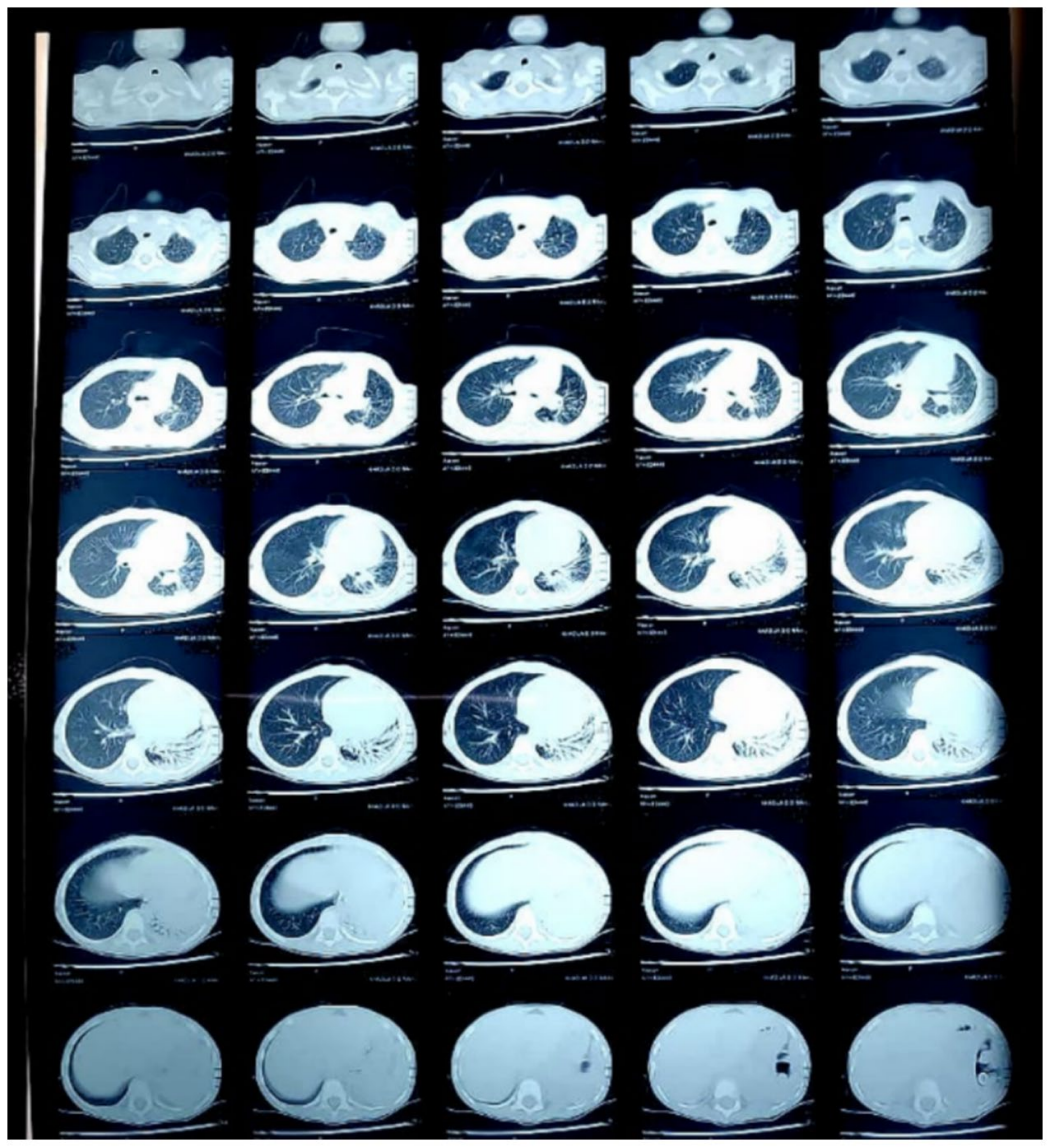
Post expulsion high resolution computed tomography showing little or no signs of consolidation.

Arterial Blood Gases and Complete Blood Count were advised (Day 01 of admission). Chest X-ray Poster-Anterior view was requested. Arterial Blood Gases and Complete Blood Count of the patient as shown in Table 1, depicts severe anemia (7.5 g/dL Hb) and hypoxemia (O₂ sat 83.6%, pO₂ 44 mmHg). Moreover, low HCO₃^−^ and pCO₂ showed mixed acid base balance disorder. Moreover, the infection was evident by complete blood picture, with marked neutrophilia, high total leukocyte count and mild thrombocytosis. Chest X-ray film was received, the radiologist reported hyperinflation of the right lung (Figure 3). The condition of the patient, along with vitals and oxygen saturation, improved the next day (Day 2 of admission). No signs of distress, no crepitus, bronchial breathing in the left lung though increased vocal fremitus was present. On the 3rd day of admission, a rather unusual event occurred: coughing up a stone of around 9 mm during one of the persistent cough episodes upon chest physiotherapy (Figures 4a,b). The patient visited for follow-up, and a High-Resolution Computed Tomography (HRCT) chest was repeated 2 days after discharge (Figure 5). Post-discharge, she remained asymptomatic, though follow-up was not maintained.

**Table 1:** Complete blood count (CBC) and arterial blood gas (ABGs) of patient showing severe hypoxemia and anemia, mixed acid–base disorder, and signs of infection.

Laboratory test	Patient’s report	Normal range
Arterial blood gases (ABGs)	O_2_ sat: 83.6% pH—7.447 pCO_2_—17.1 mmHg pO_2_—44 mmHg HCO_3_ —11 mmHg	95–100% 7.35–7.45 35–45 mmHg 80–100 mmHg 22–26 mmol/L
Complete blood count (CBC)	Hb: 7.5 g/dL TLC: 19.2 × 10^9^/L Platelets: 427 × 10^9^/L Neutrophils: 84% Lymphocytes: 13%	11.5–13.5 g/dL 5.0–15.5 × 10^9^/L 150–400 × 10^9^/L 30–60% 30–60%

## Discussion

This case of a 5-year-old girl presenting with a history of chronic cough highlights the importance of having a spectrum of differential diagnoses, especially in the absence of a history of choking (12). Differentials like asthma, pneumonia and tuberculosis should be ruled out while keeping high suspicion for foreign body. This approach enables early identification while ruling out differentials one by one, from the most common to the least common.

Foreign Body Aspiration can present symptoms like shortness of breath, cough, wheeze, and hoarseness of voice (13). Yet wheezing and hoarseness of voice were absent in this patient. Nuts, coins, fish, parts of toys, and buttons are the most common foreign bodies, while a stone was found in this patient (13, 14). In children, chronic coughs are usually associated with bacterial infection or asthma. Therefore, she kept being treated as a case of bacterial pneumonia for a span of 1 year by multiple physicians. Unresolved complaints should be investigated further to find out the cause, but a change of physician is also a hindrance to this. Patients tend to visit a second physician if symptoms persist even after the management as prescribed by the first one. In developing countries, low socioeconomic status also plays a role in the delay (15).

Intermingling etiologies and symptoms is also another hurdle in the early diagnosis of the cause of chronic cough in children. Thus, thorough history, physical examination, and investigations like Chest X-ray, High-Resolution Computed Tomography and flexible fiberoptic bronchoscopy are of great significance (9, 17). Although professional expertise alongside facility of fiberoptic bronchoscopy is limited in Pakistan and was not available at the hospital where patient was admitted. Hall hallmark of a lodged foreign body is constant lung volume during the cycle of respiration with a radiolucent foreign body in the scan. Moreover, a radiograph should be taken on expiration, which will show overinflation and hyper-lucency of the affected lung with a smaller normal lung.

In this patient, there was a history of eating disorder of consuming non-food material (PICA) and cement eating. Thus, the possibility of stone aspiration while eating gravel and mud owing to PICA is also significant. Yet on the other hand, complications like blocking the airway, traumatizing the mucosa, caustic injuries, abscesses, and in the worst case scenario, cancer can also occur (12, 14). Foreign bodies in most cases (80–90%) lodge in the right bronchus, because of is wider, more in line with the trachea, and lesser angle of convergence (13). Moreover, the location of the carina is also on the left of the midline. Thus, lodging in the left bronchus is rare.

Physiotherapy is rarely recommended alone for the removal of a foreign body. It is usually removed with the help of bronchoscopy. Cases have been reported showing delayed expulsion of forgotten foreign bodies ranging from days to months to even up to 18 years (15). Upon follow-up, no remarkable complaint was present. However, foreign body was unexpectedly coughed out in this patient upon physiotherapy, yet such modalities are contraindicated for the risk of causing subglottic obstruction. Early diagnosis can reduce the need for medical care and negate that of surgical intervention and vice versa (14). Health education should also be promoted among parents regarding the aspiration.

## Conclusion

A foreign body should be considered a cause of chronic cough in children. Although the right side is most commonly involved, occasionally, the left-sided FBA can be encountered. Despite occasional spontaneous expulsion of foreign body, bronchoscopy remains the standard of care.

## Key clinical message (KCM)

Foreign body aspiration should remain a critical differential diagnosis in any child presenting with chronic, unresolving cough—even without a history of choking. Although right-sided bronchial foreign bodies are far more common due to anatomical alignment, clinicians must remember that left-sided aspiration can also occur and may be easily missed.

## Data Availability

The original contributions presented in the study are included in the article/supplementary material, further inquiries can be directed to the corresponding author.
